# Subclinical Hyperthyroidism Could Predict Poor Outcomes in Patients With Acute Ischemic Stroke Treated With Reperfusion Therapy

**DOI:** 10.3389/fneur.2019.00782

**Published:** 2019-07-17

**Authors:** Sang-Hwa Lee, Min Uk Jang, Yerim Kim, So Young Park, Chulho Kim, Yeo Jin Kim, Jong-Hee Sohn

**Affiliations:** ^1^Department of Neurology, Chuncheon Sacred Heart Hospital, College of Medicine, Hallym University, Chuncheon, South Korea; ^2^Department of Neurology, Dongtan Sacred Heart Hospital, College of Medicine, Hallym University, Hwaseong, South Korea; ^3^Department of Neurology, Kangdong Sacred Heart Hospital, College of Medicine, Hallym University, Seoul, South Korea; ^4^Department of Endocrinology and Metabolism, Kyung Hee University Hospital, Seoul, South Korea

**Keywords:** subclinical thyroid dysfunction, subclinical hyperthyroidism, poor outcome, reperfusion therapy, acute ischemic stroke

## Abstract

**Background:** Evidence for the effect of subclinical thyroid dysfunction on the prognosis of patients suffering from acute ischemic stroke and receiving reperfusion therapy remains controversial. We aimed to investigate the association between subclinical thyroid dysfunction and the outcomes of patients with acute ischemic stroke who were treated with reperfusion therapy.

**Methods:** One hundred fifty-six consecutively recruited patients with acute ischemic stroke receiving reperfusion therapy (intravenous thrombolysis, intraarterial thrombectomy and combined intravenous thrombolysis and intraarterial thrombectomy) were included in this prospective observational study. We divided patients with subclinical thyroid dysfunction into the following 2 groups and defined a euthyroid group: subclinical hyperthyroidism (a thyroid-stimulating hormone level <0.35 μU/mL), subclinical hypothyroidism (a thyroid-stimulating hormone level >4.94 μU/mL), and a euthyroid state (0.35 μU/mL ≤ thyroid-stimulating hormone level ≤ 4.94 μU/mL). Patients with overt thyroid dysfunction were excluded. The primary outcome was functional disability at 3 months (modified Rankin Scale, mRS), and the secondary outcome was successful reperfusion. A multivariate analysis was performed to evaluate the associations between subclinical thyroid dysfunction and the primary and secondary outcomes.

**Results:** The subclinical hyperthyroidism group appeared to have poor functional outcomes, but the differences were not significant. However, compared with patients in the euthyroid state, patients with subclinical hyperthyroidism had an increased risk of poor functional outcomes at 3 months after a stroke (adjusted odds ratio [OR] 2.50, 95% confidence interval [CI] 1.01–6.14 for a mRS score of 3 to 6) and a decreased rate of successful reperfusion after reperfusion therapy (OR 0.13, 95% CI 0.04–0.43).

**Conclusion:** Subclinical hyperthyroidism may be independently associated with a poor prognosis at 3 months and unsuccessful reperfusion in patients with acute ischemic stroke receiving reperfusion therapy.

## Introduction

Previous studies have evaluated the association between subclinical thyroid dysfunction (SCTD) and the prognosis after acute ischemic stroke, with conflicting conclusions (1–5). Subclinical hypothyroidism (SCHypo) might help clinicians predict good functional outcomes after acute ischemic stroke (1, 2, 4), whereas subclinical hyperthyroidism (SCHyper) and low triiodothyronine (T3) levels appear to be risk factors for poor outcomes after stroke in some studies (6–8). According to a recent meta-analysis, patients with SCHypo may have better outcomes after an acute ischemic stroke than patients in the euthyroid state (9).

In contrast, several studies have suggested alterations in fibrinolytic activity in patients with thyroid disease (10–12). Hence, we postulated that SCTD affected the outcomes of patients with acute ischemic stroke who were treated with reperfusion therapy. Despite the results from the aforementioned studies, the effects of SCTD on the functional outcomes of patients with ischemic stroke who are treated with reperfusion therapy remain unclear.

In the present study, we aimed to investigate the associations between SCTD and the outcomes of patients with acute ischemic stroke treated with reperfusion therapy in an attempt to identify a new predictor of poor outcomes after reperfusion therapy.

## Methods

### Study Population

Using a single center registry, consecutive patients with acute ischemic stroke who received intravenous thrombolysis (IVT), intraarterial thrombectomy (IAT) and combined IVT and IAT at the Chuncheon Sacred Heart Hospital between March 2013 and March 2018 were included in this study. All patients who were eligible for reperfusion therapy underwent brain computed tomography angiography (CTA) or magnetic resonance angiography (MRA) before reperfusion therapy, according to our institutional protocol. A follow-up CTA or MRA was performed within 72 h of stroke onset. We excluded patients who had a prestroke disability (modified Rankin Scale, mRS, score >1) and whose 3-month mRS score, thyroid function tests and brain imaging data were unavailable. Additionally, we excluded patients with overt thyroid disease and patients taking medications (levothyroxine, thioamide drugs, glucocorticoid, amiodarone, thiazide, dopamine related agents, beta-blockers, and anti-epileptic drugs) that affect thyroid hormones (Database 1). Additionally, we established two separate subdatabases of Database 1 in the present study. (1) As a sensitivity analysis, we established a database (Database 2) of patients receiving IV tissue plasminogen activator (tPA; IVT and combined IVT & IAT); the patients who received IAT alone were excluded. (2) We also established a database (Database 3) of patients with large artery steno-occlusion (internal carotid artery, middle cerebral artery, anterior cerebral artery, posterior cerebral artery, basilar artery, or vertebral artery) confirmed using CTA or MRA before reperfusion therapy to evaluate the effect of SCTD on successful reperfusion (Supplemental Figure 1).

### Data Collection and Parameter Definitions

We obtained demographics, stroke risk factors, medical history, clinical characteristics and acute stroke treatment [initial National Institutes of Health Stroke Severity Scale [NIHSS] score, prestroke mRS score, ischemic stroke mechanism according to TOAST criteria, with some modifications (13), body mass index, systolic blood pressure, subtypes of reperfusion therapy and interval from onset to starting reperfusion therapy] and laboratory data from the registry database. Additionally, angiographic images of patients who were treated with reperfusion therapy were collected, and the vessel status after reperfusion therapy was graded using the Thrombolysis in Cerebral Infarction (TICI) grading criteria (14).

### Measurement of Thyroid Function

Blood samples were obtained within 18 h of stroke onset to assess TSH, fT4 and total T3 levels using a chemiluminescence-based immunoassay (ARCHITECT TSH). Considering the diurnal TSH variation, we collected blood samples in the late morning and during the day (09:00 to 15:00) (15). The normal levels of TSH for our laboratory ranged from 0.35 to 4.94 μU/mL. We divided the patients with SCTD into the following 3 groups: the SCHyper group (TSH <0.35 μU/mL), the SCHypo group (TSH > 4.94 μU/mL), and the euthyroid group (0.35 μU/mL ≤ TSH ≤ 4.94 μU/mL) with normal fT4 and total T3 levels.

### Outcome Assessments

The primary outcome measure was a poor functional outcome (mRS score of 0–2 vs. 3–6) in the euthyroid, SCHypo and SCHyper groups. The secondary outcome measure was the proportion of patients with unfavorable functional outcomes (mRS score of 0–1 vs. 2–6) and successful reperfusion. Successful reperfusion was defined as TICI grades 2b to 3 in patients receiving IAT and complete revascularization on a follow-up MRA in patients receiving IVT alone. Symptomatic hemorrhagic transformation after reperfusion therapy was defined according to the definition of the European Cooperative Acute Stroke Study (16). The volume of the ischemic lesion confirmed by DWI was calculated using Medical Image Processing and Visualization software (version 7.3.0, National Institutes of Health, Bethesda, MD). The IV administered tPA dose was 0.9 mg/kg in all subjects.

### Statistical Analysis

Summary statistics are presented as the number of subjects (percentage) for categorical variables and as the means ± SD or medians (interquartile ranges, IQR) for continuous variables. Differences between groups were compared using Pearson's chi-square test or Fisher's exact test for categorical variables and ANOVA or the Kruskal-Wallis test for continuous variables, as appropriate.

Regarding the primary and secondary outcome measures, the euthyroid, SCHypo and SCHyper groups were compared using Pearson's chi-square test, and the independent effects of SCTD on those outcome measures were analyzed using binary logistic regression analyses (using Databases 1, 2, and 3). The variables that were adjusted in the multivariate analysis were selected based on *p*-values < 0.1 in comparisons according to the SCTD group and clinically plausible associations with each outcome variable. A sensitivity analysis was performed using Database 2 to determine the effect of SCTD on functional outcomes in only patients treated with IVT, since IAT could increase the heterogeneity in relation to reperfusion therapy. A multivariate analysis was performed using Database 3 to determine the effect of SCTD on the successful reperfusion rate. Crude and adjusted odd ratios (ORs) and 95% confidence intervals (CIs) were estimated. All analyses were performed with IBM SPSS version 21.0 software (IBM Corporation, Armonk, NY, USA).

## Results

Among the 1,360 consecutively enrolled patients with stroke, 156 patients (93 males; mean age 70.3 ± 11.6 years) with acute ischemic stroke who received reperfusion therapy were included in the study (Database 1) over the study period. Among these patients, 104 (66.7%) had TSH levels in the normal range (euthyroid group), 15 (9.6%) had TSH levels above the normal range (SCHypo group), and 37 (23.7%) had TSH levels below the normal range (SCHyper group). The median NIHSS score was 10 (IQR 6–16), and the mean ischemic lesion volume was 34.5 cm^3^ (±70.0). Among the 156 patients, 125 received IV tPA (Database 2) and 117 had large artery steno-occlusion at the time of reperfusion therapy (Database 3). The overall proportions of patients with an unfavorable functional outcome (mRS score of 2 to 6) and a poor functional outcome (mRS score of 3 to 6) were 74.4% (*n* = 116) and 53.2% (*n* = 83), respectively. Additionally, 17 (10.9%) patients experienced symptomatic hemorrhagic transformation. Compared with the euthyroid and SCHyper groups, the SCHypo group was more likely to have diabetes mellitus and earlier visits after stroke onset. Among the patients receiving IV tPA (IVT and combined IVT & IAT), patients in the SCHypo group were more likely have lower total cholesterol levels, low-density lipoprotein levels and systolic blood pressure than the other groups. Generally, the stroke characteristics and demographics were not different between the three SCTD groups (Table 1; Supplemental Table 1).

**Table 1:** Baseline characteristics of all patients receiving reperfusion therapy stratified according to SCTD status.

	**Euthyroid group**	**SCHypo group**	**SCHyper group**	***p*-value**
	**(*n* = 104)**	**(*n* = 15)**	**(*n* = 37)**	
Age, years (SD)	70.9 (10.8)	74.4 (10.7)	67.1 (13.7)	0.09^‡^
Male, (%)	63 (60.6)	6 (40.0)	24 (64.9)	0.27^*^
BMI, kg/m^2^ (SD)	24.6 (4.0)	21.9 (4.7)	24.2 (3.6)	0.052^‡^
Initial NIHSS score, (IQR)	10 (6–16)	11 (7–17)	11 (6–15)	0.62^§^
NIHSS score at discharge, (IQR)	2 (0–5)	1 (1–3)	3 (1–9)	0.20^§^
Stroke subtypes, (%)				0.15^†^
SVO	9 (8.7)	0 (0.0)	5 (13.5)	
LAA	31 (29.8)	1 (6.7)	14 (37.8)	
CE	44 (42.3)	9 (60.0)	12 (32.4)	
Others	20 (19.2)	5 (33.3)	6 (16.2)	
Stroke history, (%)	26 (25.0)	5 (33.3)	10 (27.0)	0.82^*^
CHD, (%)	13 (12.5)	1 (6.7)	4 (10.8)	0.87^†^
Hypertension, (%)	63 (60.6)	7 (46.7)	17 (45.9)	0.25^*^
Diabetes mellitus, (%)	38 (36.5)	2 (13.3)	7 (18.9)	0.047^*^
Hyperlipidemia, (%)	18 (17.3)	2 (13.3)	5 (13.5)	0.83^*^
Current smoking, (%)	32 (20.2)	1 (6.7)	8 (21.6)	0.42^*^
AF, (%)	39 (37.5)	10 (66.7)	14 (37.8)	0.097^*^
History of antithrombotics, (%)	43 (41.3)	8 (53.3)	12 (32.4)	0.37^*^
Interval from onset to visit, min (SD)	155.0 (202.0)	71.7 (53.5)	121.1 (143.8)	0.03^‡^
Reperfusion therapy, (%)				0.99^†^
IVT	54 (61.5)	10 (66.7)	25 (67.6)	
IAT	15 (14.4)	2 (13.3)	4 (10.8)	
Combined	25 (24.0)	3 (20.0)	8 (21.6)	
SHT, (%)	10 (9.6)	2 (13.3)	5 (13.5)	0.65^†^
Creatinine, mg/dL (SD)	1.03 (0.66)	0.84 (0.17)	1.01 (0.54)	0.53^‡^
LDL, mg/dL (SD)	91.9 (29.0)	112.5 (56.3)	96.7 (30.3)	0.07^‡^
HbA1c, % (SD)	6.3 (1.4)	6.1 (1.3)	5.9 (0.9)	0.31^‡^
PT, INR (SD)	1.04 (0.19)	1.02 (0.10)	1.06 (0.18)	0.74^‡^
SBP, mmHg (SD)	143.0 (29.0)	153.3 (29.2)	155.6 (24.4)	0.04^‡^
Ischemic lesion volume, cm^3^ (IQR)	4.7 (0.9–30.1)	2.8 (0.4–30.3)	4.7 (0.7–25.9)	0.80^§^

*SCTD, subclinical thyroid dysfunction; BMI, body mass index; NIHSS, National Institutes of Health Stroke Scale; SVO, small vessel occlusion; LAA, large artery atherosclerosis; CE, cardioembolism; AF, atrial fibrillation; IVT, intravenous thrombolysis; IAT, intraarterial thrombectomy; SHT, symptomatic hemorrhagic transformation; LDL, low-density lipoprotein; HbA1c, glycated hemoglobin; PT, prothrombin time; INR, international normalized ratio; CRP, C-reactive protein; SBP, systolic blood pressure *.

* *Calculated using the chi-square test*.

† *Calculated using Fisher's exact test*.

‡ *Calculated using ANOVA*.

§ *Calculated using the Kruskal-Wallis test*.

**Figure 1:**
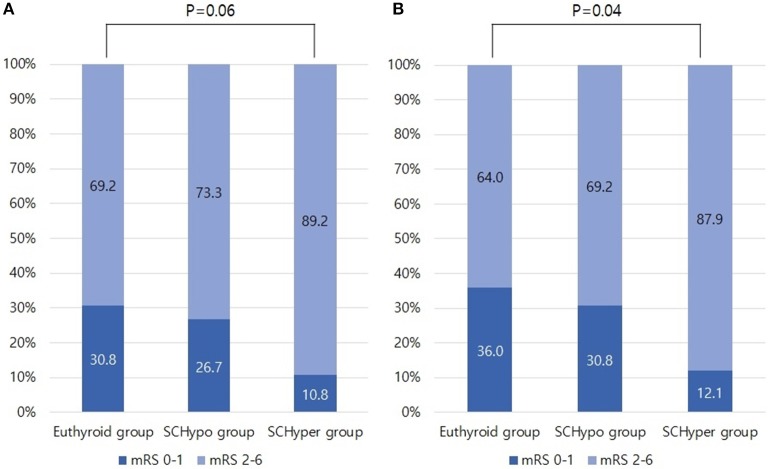
Proportion of unfavorable functional outcomes in the SCTD groups, defined as 3-month mRS scores of 2 to 6. Panel **(A)** shows the proportion of patients with unfavorable functional outcomes in Database 1 (*n* = 156). Panel **(B)** shows the proportion of patients with unfavorable functional outcomes in Database 2 (*n* = 125). mRS, modified Rankin Scale; SCTD, subclinical thyroid dysfunction; IV tPA, intravenous tissue plasminogen activator.

The proportion of unfavorable functional outcomes tended to be higher in the SCHyper group than in the euthyroid and SCHypo groups (69.1% for the euthyroid group, 73.3% for the SCHypo group and 89.2% for the SCHyper group, *p* = 0.06, Figure 1A). Among the 125 patients receiving IV tPA, a higher proportion of patients in the SCHyper group experienced unfavorable functional outcomes than patients in the euthyroid and SCHypo groups (64.0% for the euthyroid group, 69.3% for the SCHypo group, 87.9% for the SCHyper group, *p* = 0.04, Figure 1B). Symptomatic hemorrhagic transformation did not differ according to SCTD status (*p* = 0.65).

According to the multivariate analysis, compared to the euthyroid and SCHypo groups, the SCHyper group had an increased risk of poor functional outcomes at 3 months (adjusted OR 2.50, 95% CI 1.01–6.14 for an mRS score of 3 to 6, Table 2). Among patients receiving IV tPA, patients in the SCHyper group had an increased risk of poor functional outcomes at 3 months compared to patients in the euthyroid and SCHypo groups (Supplemental Table 2).

**Table 2:** Multivariate analysis of the associations between SCTD and poor functional outcomes at 3 months.

	**Adjusted OR**	**95% CI**	***p*-value**
Age	1.01	0.97–1.04	0.78
Male	0.59	0.28–1.25	0.17
NIHSS score	1.14	1.06–1.22	<0.001
DM	1.43	0.65–3.16	0.38
AF	0.70	0.32–1.56	0.39
SBP	1.00	0.99–1.01	0.95
Interval from onset to visit	1.001	0.999–1.003	0.39
Euthyroid group	Reference		
SCHypo group	0.98	0.29–3.34	0.97
SCHyper group	2.50	1.01–6.14	0.047

*SCTD, subclinical thyroid dysfunction; mRS, modified Rankin Scale; OR, odds ratio; CI, confidence interval; DM, diabetes mellitus; AF, atrial fibrillation; SBP, systolic blood pressure; SCHypo, subclinical hypothyroidism; SCHyper, subclinical hyperthyroidism*.

**Figure 2:**
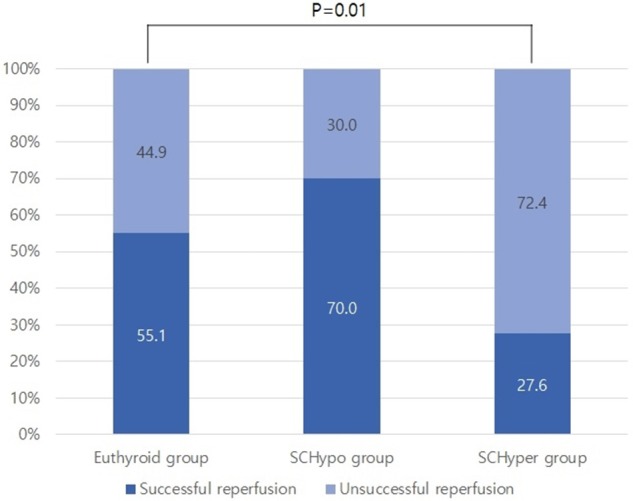
The rate of successful reperfusion in the SCTD groups. SCTD, subclinical thyroid dysfunction; SCHyper, subclinical hyperthyroidism; SCHypo, subclinical hypothyroidism.

Among the 117 patients with large artery steno-occlusion before reperfusion therapy, the rate of successful reperfusion was lower among patients in the SCHyper group than among patients in the euthyroid and SCHypo groups (70.0% for the SCHypo group, 55.1% for the euthyroid group and 27.6% for the SCHyper group, *p* = 0.01, Figure 2). The baseline characteristics of the 117 patients with large artery steno-occlusion before reperfusion therapy stratified according to SCTD status are shown in Supplemental Table 3. Based on the results of the multivariate analysis, the SCHyper group had a decreased chance of successful reperfusion compared to the euthyroid and SCHypo groups (adjusted OR 0.13, 95% CI 0.04–0.43, Table 3).

**Table 3:** Multivariate analysis of the association between SCTD and successful reperfusion.

	**Adjusted OR**	**95% CI**	***p*-value**
Age	0.96	0.92–1.00	0.048
Male	0.88	0.34–2.29	0.80
NIHSS score	1.10	1.02–1.19	0.02
DM	0.54	0.21–1.40	0.21
AF	0.20	0.07–0.55	0.002
SBP	1.02	0.999–1.04	0.07
Euthyroid group	Reference		
SCHypo group	0.85	0.16–4.42	0.84
SCHyper group	0.13	0.04–0.43	0.001

*SCTD, subclinical thyroid dysfunction; OR, odds ratio; CI, confidence interval; DM, diabetes mellitus; AF, atrial fibrillation; SBP, systolic blood pressure; SCHypo, subclinical hypothyroidism; SCHyper, subclinical hyperthyroidism*.

## Discussion

The main findings of this study are listed below. (1) The SCHyper group receiving reperfusion therapy tended to have worse functional outcomes, particularly in patients receiving IV tPA. (2) SCHyper was independently associated with poor functional outcomes at 3 months. (3) SCHyper was a potential risk factor for a decrease in the successful reperfusion of large vessel steno-occlusion after reperfusion therapy.

Thyroid hormone levels are often altered in patients with ischemic stroke, with approximately 36% of patients with ischemic stroke presenting TSH levels (17). Hence, the relationship between TSH levels and the prognosis after ischemic stroke is a matter of increasing interest. Although several studies have examined the association between TSH and stroke outcomes (2, 4, 8, 18), the effects of SCTD on functional outcomes and successful reperfusion in patients with acute ischemic stroke receiving reperfusion therapy have not been investigated. The results of our study will allow us to better predict the outcomes for patients with acute ischemic stroke presenting with SCTD prior to reperfusion therapy.

Interestingly, we observed an effect of SCTD on the rate of reperfusion. Coagulability among patients with SCTD has long been a topic of debate (19). Hyperthyroidism might be related to a hypercoagulable state (20, 21), and SCHyper decreases fibrinolytic activity (19). Compared with the euthyroid controls, patients with hyperthyroidism and SCHyper present significantly increased levels of fibrinogen, factor IX, von Willebrand factor, antithrombin III and plasminogen activator inhibitor-1 and decreased levels of factor X, tissue-type plasminogen activator, and tissue factor pathway inhibitor antigen, suggesting a reduced fibrinolytic capacity (22, 23). Although the levels of coagulation factors were unavailable in this study, the possible mechanism described above might explain the unsuccessful reperfusion in patients in the SCHyper group. In addition, hyperthyroidism may be associated with increased arterial stiffness (24, 25). Although the association between thyroid dysfunction and arterial stiffness remains controversial, SCHyper potentially affects impaired vascular elasticity, thereby reducing the reperfusion rate after reperfusion therapy.

Our result showed that SCHyper was associated with unsuccessful reperfusion and could increase the unfavorable and poor functional outcomes. Since the reperfusion rate potentially exerts a profound effect on outcomes 3 months after stroke, we should be cautious when generalizing our results that SCTD affects the functional outcomes after reperfusion therapy. The interaction analysis showed that the association between SCTD and poor functional outcome was significantly mediated by the reperfusion rate in our database (*p for interaction* 0.01, data were not shown). Additionally, subgroup analysis revealed that SCHyper had a disparity of association for poor functional outcomes according to reperfusion status (Supplemental Table 4). This result could explain the greater effect of reperfusion status on functional outcome in stroke patients with no SCTD. Nonetheless, the goal of the present study was to investigate the impact of underestimated SCTD in patients with ischemic stroke who underwent reperfusion therapy. We carefully suggested that our results may allow clinicians to use SCTD as a new prognostic marker for patients with ischemic stroke.

In the present study, the initial stroke severity and ischemic lesion volume did not differ according to SCTD status. In contrast, TSH levels were associated with a lower stroke severity and better outcomes at discharge due to a preconditioning effect of TSH in a previous study (4). This previous study examined a relatively large sample size (*n* = 731), but it did not investigate the ischemic lesion volume. Since the sample sizes of the SCHypo group and the total population, including patients with a severe stroke (median NIHSS score = 10, mean ischemic lesion volume = 34.5 cm^3^), who received reperfusion therapy were small in this study, the effect of TSH levels on the outcomes of patients in the acute phase of stroke might be attenuated. However, the results of the effect of SCTD on delayed functional outcomes in the present study were consistent with those of previous studies. Hence, we cautiously suggest that the effects of TSH on the acute and delayed phases of stroke might different in patients with a severe stroke. Further studies on this issue are needed to confirm the different effects of TSH on patients stratified according to the stroke phase and initial stroke severity.

Regarding the stroke subtypes, cardioembolic stroke did not differ in patients stratified according to the SCTD status in the present study. According to several previous studies, SCHyper increases the risks of atrial fibrillation and cardioembolic stroke (20), which were associated with worse outcomes than other stroke subtypes (26). A potential explanation for this result is the early detection of thyroid hormone levels in the present study. A large cohort study reported a significantly increased risk of atrial fibrillation in patients with SCHyper after 12 months (27). In the present study, we only included patients with a history of newly detected atrial fibrillation at hospitalization. Therefore, we must conduct long-term observations of patients using follow-up electrocardiography. However, due to the small sample size of the SCHyper group, the possibility of a decreased association with atrial fibrillation should also be considered.

Although this novel study showed an effect of SCTD on functional outcomes and successful reperfusion in patients with stroke who were treated with reperfusion therapy, several limitations should be considered. First, this study was performed at a single center on a small sample size, although we used a prospective database. The smaller sample size of the SCHypo group compared with the two SCTD groups might increase the imbalance in baseline stroke characteristics, particularly in patients with different stroke subtypes [large artery occlusion (LAA) vs. cardioembolism (CE)]. Further studies with larger sample sizes should be performed. Second, the initial TSH level might have been altered due to stroke recovery and the administration of iodinated radio-contrast agents for CTA. A large cerebral infarction is associated with alterations in thyroid hormone levels after 3 days (28). Additionally, TSH levels increase significantly 3–5 days after iodine administration (29). Since we collected blood samples within 18 h of stroke onset, we postulated that the TSH levels in this study reflect the prestroke thyroid status and were not influenced by acute stroke or iodinated contrast agents. Similarly, blood samples were collected at different times, and the influence of SCTD on the reperfusion rate is questionable. Increasing TSH levels and alterations in the levels of other thyroid hormones have been detected at 24 h, 2 days and 5 days after stroke onset (28, 30, 31). Hence, we assumed that the variation in the sample collection time would not affect the observed results. In addition, a blood sample was collected from 127 of the 156 patients before reperfusion therapy in the emergency department (81.4%). We believe that this proportion of early sampling increased the validity of the findings from our study. Third, heterogeneity in reperfusion therapies (e.g., add-on IAT) might affect the validity of the results. However, the proportions of patients receiving reperfusion therapy were not different among the three SCTD groups. Moreover, the positive correlation between unfavorable functional outcomes and SCHyper in patients receiving IV tPA supported the robustness of our main findings. Fourth, the durations of SCTD in each subject were not available in this study. An increasing duration of thyroid dysfunction is strongly correlated with a poor prognosis (32). The next study on this issue should also consider the duration of thyroid dysfunction. Finally, our study period may not be appropriate because the acute stroke treatment was dramatically revised by trials published in 2015. However, we included subjects who only received intraarterial mechanical thrombectomy, but not “*thrombolysis*,” in the present study.

## Conclusions

This study revealed significant associations of SCHyper with a worse prognosis and unsuccessful reperfusion in patients with acute ischemic stroke receiving reperfusion therapy. Our findings contribute to a better understanding of the adverse effects of SCHyper. The underlying mechanisms and prognostic predictors of SCTD require confirmation in further studies.

## Data Availability

The raw data supporting the conclusions of this manuscript will be made available by the authors, without undue reservation, to any qualified researcher.

## Ethics Statement

Because the anonymity of study subjects was maintained and patients had minimal risks, the collection of data without informed consent was approved by the local institutional review board (IRB) of the Chuncheon Sacred Heart Hospital before the study commenced (IRB No. 2018-06-002).

## Author Contributions

S-HL: study design, clinical and imaging data acquisition, data analysis and interpretation, and primary writing. J-HS: study design, clinical and imaging data acquisition, data interpretation, critical revision of the manuscript for important intellectual content, and supervision of the study. MJ, SP, YK, YJK, and CK: data acquisition and critical revision of the manuscript for intellectual content.

### Conflict of Interest Statement

The authors declare that the research was conducted in the absence of any commercial or financial relationships that could be construed as a potential conflict of interest.
