# Supplementary material for: Subclinical Hyperthyroidism Could Predict Poor Outcomes in Patients With Acute Ischemic Stroke Treated With Reperfusion Therapy
Source: Front Neurol. 2019 Jul 17;10:782. doi: 10.3389/fneur.2019.00782 (PMC6652740; doi:10.3389/fneur.2019.00782)

**Online Supplement**

Supplemental Table 1. Baseline characteristics according to SCTD receiving IV tPA. (Database 2, n=125)

|  | Euthyroid group  (n=89) | SCHypo group  (n=13) | SCHyper group  (n=23) | P-value |
| --- | --- | --- | --- | --- |
| Age, year (SD) | 69.9 (10.9) | 73.0 (10.8) | 65.5 (13.5) | 0.08^‡^ |
| Male, (%) | 53 (59.6) | 6 (46.2) | 23 (69.7) | 0.32^*^ |
| BMI, kg/m^2^ (SD) | 35.9 (103.3) | 22.5 (4.4) | 24.6 (3.5) | 0.74^‡^ |
| Initial NIHSS, (IQR) | 10 (6-15) | 11 (7-15) | 10 (6-14) | 0.82^§^ |
| NIHSS at discharge, (IQR) | 2 (0-4) | 1 (1-2) | 2 (1-5) | 0.20^§^ |
| Stroke subtypes, (%) |  |  |  | 0.12^†^ |
| SVO | 9 (10.1) | 0 (0.0) | 5 (15.2) |  |
| LAA | 28 (31.5) | 1 (737) | 14 (42.4) |  |
| CE | 34 (38.2) | 7 (53.8) | 9 (27.3) |  |
| Others | 18 (20.2) | 5 (38.5) | 5 (15.2) |  |
| Stroke history, (%) | 23 (25.8) | 4 (30.8) | 9 (27.3) | 0.95^*^ |
| CHD, (%) | 10 (11.2) | 1 (7.7) | 2 (6.1) | 0.81^†^ |
| Hypertension, (%) | 54 (60.7) | 6 (46.2) | 15 (45.5) | 0.25^*^ |
| Diabetes mellitus, (%) | 31 (34.8) | 2 (15.4) | 6 (18.2) | 0.10^*^ |
| Hyperlipidemia, (%) | 15 (16.9) | 2 (15.4) | 4 (12.1) | 0.81^*^ |
| Current smoking, (%) | 21 (23.6) | 1 (7.7) | 8 (24.2) | 0.46^*^ |
| AF, (%) | 31 (34.8) | 8 (61.5) | 10 (30.3) | 0.12^*^ |
| History of antithrombotics, (%) | 37 (41.6) | 7 (53.8) | 10 (30.3) | 0.32^*^ |
| Interval from onset to visit, min (SD) | 129.7 (190.2) | 76.8 (56.0) | 76.7 (49.6) | 0.19^‡^ |
| Interval from visit to needle time, min (SD) | 42.9 (29.1) | 45.8 (27.9) | 47.5 (38.3) | 0.76^‡^ |
| SHT, (%) | 8 (9.0) | 2 (15.4) | 4 (12.1) | 0.68^†^ |
| WBC, x1000/µL, (SD) | 7.6 (3.7) | 7.6 (2.4) | 7.3 (2.6) | 0.89^‡^ |
| Total cholesterol, mg/dL, (SD) | 153.5 (33.3) | 176.9 (56.1) | 166.1 (37.8) | 0.048^‡^ |
| Creatinine, mg/dL (SD) | 1.04 (0.71) | 0.80 (0.14) | 1.00 (0.64) | 0.45^‡^ |
| Hemoglobin, g/dL (SD) | 13.4 (1.9) | 14.2 (1.9) | 13.5 (1.9) | 0.41^‡^ |
| Platelet, x1000/µL (SD) | 227.9 (86.2) | 246.9 (44.9) | 220.8 (71.5) | 0.61^‡^ |
| LDL, mg/dL (SD) | 89.7 (28.1) | 116.2 (54.8) | 98.1 (31.4) | 0.02^‡^ |
| HbA1c, % (SD) | 6.3 (1.5) | 6.1 (1.4) | 6.0 (0.9) | 0.47^‡^ |
| PT, INR (SD) | 1.03 (0.17) | 1.00 (0.09) | 1.07 (0.19) | 0.52^‡^ |
| CRP, mg/L (SD) | 9.91 (24.30) | 3.10 (2.57) | 12.09 (43.15) | 0.64^‡^ |
| Initial random glucose, mg/dL (SD) | 135.6 (57.6) | 118.5 (45.5) | 124.8 (33.5) | 0.39^‡^ |
| SBP, mmHg (SD) | 142.1 (29.6) | 159.2 (26.6) | 154.8 (25.2) | 0.03^‡^ |
| T3, ng/dL (SD) | 79.0 (14.8) | 81.3 (29.0) | 80.1 (18.3) | 0.88^‡^ |
| fT4, ng/dL (SD) | 1.0.3 (0.17) | 1.05 (0.18) | 1.06 (0.14) | 0.68^‡^ |
| TSH, µIU/mL (SD) | 1.27 (0.86) | 6.21 (1.35) | 0.24 (0.12) | <0.001^‡^ |
| Ischemic lesion volume, cm^3^ (IQR) | 4.4 (0.8-22.7) | 1.7 (0.3-22.4) | 4.7 (0.7-25.9) | 0.64^§^ |

Abbreviations: SCTD, subclinical thyroid dysfunction; IV tPA, intravenous tissue plasminogen activator; BMI, body mass index; NIHSS, National Institute of Health Stroke Scale; SVO, small vessel occlusion; LAA, large artery atherosclerosis; CE, cardioembolism; AF, atrial fibrillation; IVT, intravenous thrombolysis; IAT, intraarterial thrombectomy; SHT, symptomatic hemorrhagic transformation; WBC, white blood cell; LDL, low density lipoprotein; HbA1c, glycated hemoglobin; PT, prothrombin time; INR, international normalized ratio; CRP, C-reactive protein; SBP, systolic blood pressure; T3, triiodothyronine; fT4, free thyroxine; TSH, thyroid stimulating hormone

^*^ Calculated by the chi-square test

^†^ Calculated by Fisher’s exact test

^‡^ Calculated by ANOVA

^§^ Calculated by the Kruskal-Wallis test

Supplemental Table 2. Multivariable analysis: association between SCTD and poor functional outcomes at 3 months in patients receiving IV tPA

|  | Adjusted OR | 95% CI | P-value |
| --- | --- | --- | --- |
| Age | 1.03 | 0.99-1.07 | 0.16 |
| Male | 0.67 | 0.29-1.54 | 0.34 |
| NIHSS | 1.18 | 1.09-1.27 | <0.001 |
| DM | 1.74 | 0.72-4.23 | 0.22 |
| LDL | 1.01 | 0.998-1.02 | 0.09 |
| SBP | 1.001 | 0.99-1.02 | 0.86 |
| Euthyroid group | reference | |  |
| SCHypo group | 0.91 | 0.23-3.63 | 0.89 |
| SCHyper group | 2.82 | 1.04-7.67 | 0.04 |

Abbreviations: SCTD, subclinical thyroid dysfunction; IV tPA, intravenous tissue plasminogen activator; mRS, modified Rankin Scale; OR, odd ratio; CI, confidence interval; NIHSS, National Institute of Health Stroke Scale; DM, diabetes mellitus; LDL, low density lipoprotein; SBP, systolic blood pressure; SCHypo, subclinical hypothyroidism; SCHyper, subclinical hyperthyroidism

Supplemental Table 3. Baseline characteristics according to SCTD who had large artery occlusion (Database 3, n=117).

|  | Euthyroid group  (n=78) | SCHypo group  (n=10) | SCHyper group  (n=29) | p-value |
| --- | --- | --- | --- | --- |
| Age, year (SD) | 71.7 (10.7) | 77.9 (7.50) | 68.7 (14.2) | 0.09^‡^ |
| Male, (%) | 49 (62.8) | 4 (40.0) | 18 (62.1) | 0.39^*^ |
| BMI, kg/m^2^ (SD) | 37.3 (110.4) | 20.7 (5.0) | 24.0 (3.6) | 0.73^‡^ |
| Initial NIHSS, (IQR) | 12 (7-17) | 13.5 (11-18) | 12 (8-16) | 0.66^§^ |
| NIHSS at discharge, (IQR) | 2 (0-6) | 1 (1-7) | 3 (1-9) | 0.22^§^ |
| Stroke subtypes, (%) |  |  |  | 0.18^†^ |
| SVO | 2 (2.6) | 0 (0.0) | 1 (3.4) |  |
| LAA | 26 (33.3) | 0 (0.0) | 12 (41.4) |  |
| CE | 38 (48.7) | 8 (80.0) | 11 (37.9) |  |
| Others | 12 (15.4) | 2 (20.2) | 5 (17.2) |  |
| Stroke history, (%) | 22 (28.2) | 3 (30.0) | 9 (31.0) | 0.95^*^ |
| CHD, (%) | 11 (14.1) | 1 (10.0) | 4 (13.8) | 1.00^†^ |
| Hypertension, (%) | 49 (62.8) | 4 (40.0) | 15 (51.7) | 0.29^*^ |
| Diabetes mellitus, (%) | 29 (37.2) | 1 (10.0) | 6 (20.7) | 0.09^*^ |
| Hyperlipidemia, (%) | 11 (14.1) | 1 (10.0) | 5 (17.2) | 0.84^†^ |
| Current smoking, (%) | 16 (20.5) | 1 (10.0) | 7 (24.1) | 0.67^*^ |
| AF, (%) | 34 (43.6) | 9 (90.0) | 13 (44.8) | 0.02^*^ |
| History of antithrombotics, (%) | 35 (44.9) | 5 (50.0) | 11 (37.9) | 0.80^*^ |
| Interval from onset to visit, min (SD) | 173.7 (227.6) | 72.1 (52.0) | 129.9 (159.9) | 0.26^‡^ |
| Reperfusion therapy, (%) |  |  |  | 0.92^*^ |
| IVT | 38 (48.7) | 5 (50.0) | 17 (58.6) |  |
| IAT | 15 (19.2) | 2 (20.2) | 4 (13.8) |  |
| Combined | 25 (32.1) | 3 (30.3) | 8 (27.6) |  |
| SHT | 8 (10.3) | 2 (20.0) | 4 (13.8) | 0.49^†^ |
| Successful reperfusion (%) | 43 (55.1) | 7 (70.0) | 8 (27.6) | 0.01^*^ |
| WBC, x1000/µL, (SD) | 7.52 (2.99) | 7.41 (1.59) | 7.49 (2.69) | 0.99^‡^ |
| Total cholesterol, mg/dL, (SD) | 37.3 (4.2) | 46.1 (14.6) | 29.6 (5.5) | 0.72^‡^ |
| Creatinine, mg/dL (SD) | 1.04 (0.75) | 0.85 (0.17) | 1.04 (0.60) | 0.71^‡^ |
| Hemoglobin, g/dL (SD) | 13.6 (1.8) | 13.9 (1.5) | 13.9 (1.6) | 0.66^‡^ |
| Platelet, x1000/µL (SD) | 227.5 (89.7) | 252.6 (49.2) | 217.3 (65.7) | 0.50^‡^ |
| LDL, mg/dL (SD) | 92.1 (28.6) | 105.5 (50.7) | 94.9 (24.7) | 0.41^‡^ |
| HbA1c, % (SD) | 6.3 (1.3) | 6.1 (1.5) | 6.0 (1.0) | 0.56^‡^ |
| PT, INR (SD) | 1.03 (0.20) | 1.05 (0.11) | 1.06 (0.15) | 0.85^‡^ |
| CRP, mg/L (SD) | 8.49 (21.11) | 7.52 (15.41) | 14.34 (46.09) | 0.63^‡^ |
| Initial random glucose, mg/dL (SD) | 135.9 (52.8) | 104.9 (23.3) | 124.1 (36.3) | 0.11^‡^ |
| SBP, mmHg (SD) | 143.9 (28.6) | 143.0 (23.6) | 160.6 (27.6) | 0.02^‡^ |
| T3, ng/dL (SD) | 77.6 (15.5) | 80.7 (33.9) | 78.1 (19.1) | 0.89^‡^ |
| fT4, ng/dL (SD) | 1.03 (0.17) | 1.10 (0.19) | 1.07 (0.16) | 0.46^‡^ |
| TSH, µIU/mL (SD) | 1.20 (0.83) | 6.26 (1.58) | 0.23 (0.12) | <0.001^‡^ |
| Ischemic lesion volume, cm^3^ (IQR) | 7.2 (1.9-43.7) | 7.2 (1.7-38.3) | 10.0.0 (1.3-33.4) | 0.96^§^ |

Abbreviations: SCTD, subclinical thyroid dysfunction; BMI, body mass index; NIHSS, National Institute of Health Stroke Scale; SVO, small vessel occlusion; LAA, large artery atherosclerosis; CE, cardioembolism; AF, atrial fibrillation; IVT, intravenous thrombolysis; IAT, intraarterial thrombectomy; SHT, symptomatic hemorrhagic transformation; WBC, white blood cell; LDL, low density lipoprotein; HbA1c, glycated hemoglobin; PT, prothrombin time; INR, international normalized ratio; CRP, C-reactive protein; SBP, systolic blood pressure; T3, triiodothyronine; fT4, free thyroxine; TSH, thyroid stimulating hormone

^*^ Calculated by the chi-square test

^†^ Calculated by Fisher’s exact test

^‡^ Calculated by ANOVA

^§^ Calculated by the Kruskal-Wallis test

Supplemental table 4. Multivariable analysis: association between SCTD and poor functional outcomes (mRS score of 3-6) at 3 months according to reperfusion status

|  | Unsuccessful reperfusion  (TICI grade 0-2a, n=59) | | Successful reperfusion  (TICI grade 2b-3, n=58) | |
| --- | --- | --- | --- | --- |
|  | Adjusted OR | 95% CI | Adjusted OR | 95% CI |
| Age | 0.999 | 0.93-1.08 | 0.96 | 0.90-1.01 |
| Male | 0.35 | 0.06-1.95 | 0.35 | 0.10-1.26 |
| NIHSS score | 1.25 | 1.07-1.46 | 1.07 | 1.95-1.19 |
| DM | 1.22 | 0.20-7.34 | 0.62 | 0.16-2.37 |
| AF | 0.78 | 0.11-5.76 | 0.44 | 0.11-1.72 |
| SBP | 0.98 | 0.94-1.02 | 0.999 | 0.98-1.02 |
| Euthyroid group | reference | | reference | |
| SCHypo group | 1.31 | 0.06-27.99 | 0.40 | 0.06-2.58 |
| SCHyper group | 10.38 | 1.32-81.59 | 0.10 | 0.01-0.79 |

Abbreviations: SCTD, subclinical thyroid dysfunction; mRS, modified Rankin Scale; OR, odd ratio; CI, confidence interval; DM, diabetes mellitus; SBP, systolic blood pressure; SCHypo, subclinical hypothyroidism; SCHyper, subclinical hyperthyroidism

Supplemental figure 1. Study flow chart


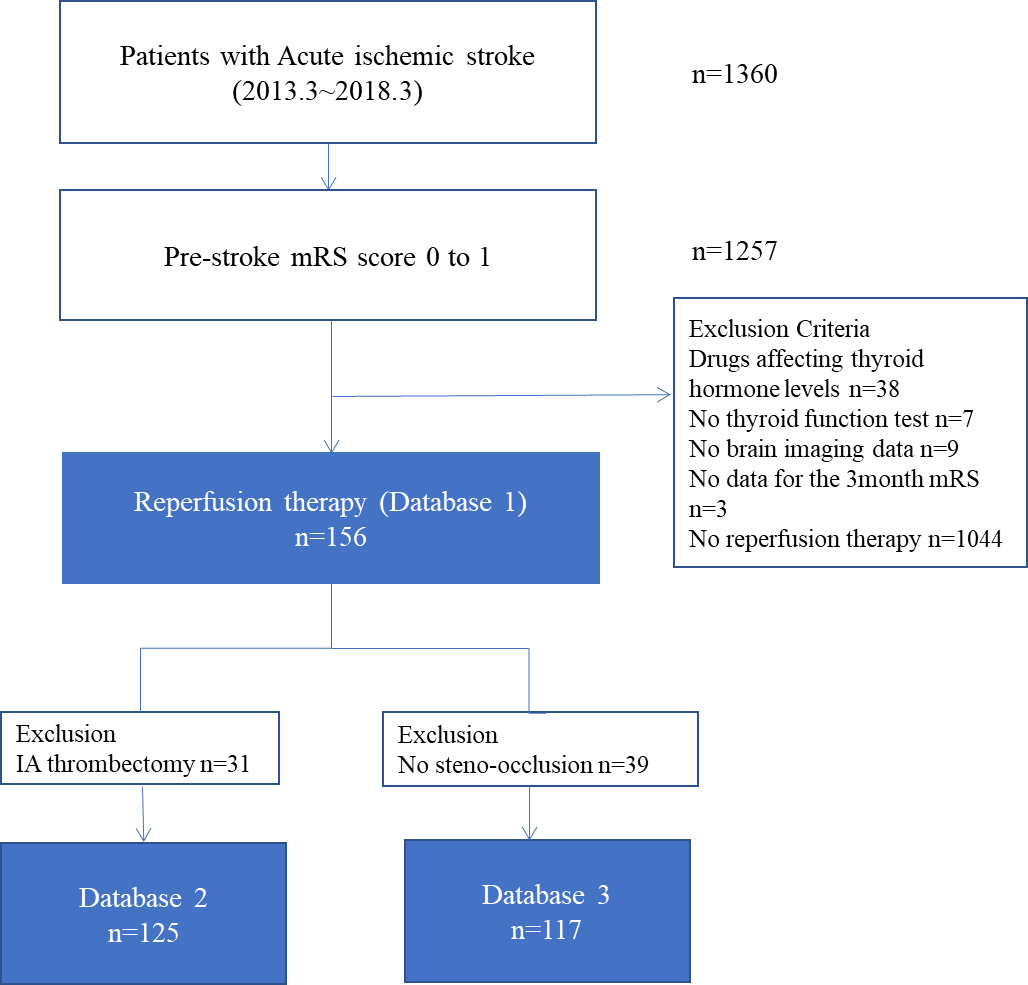

Supplement: Supplementary file 1 [file Table_1.DOCX]
